# Genome-Wide Characterization of *WRKY* Gene Family in *Camellia chekiangoleosa* Identifies Potential Regulatory Components in Pigment Biosynthesis Pathways

**DOI:** 10.3390/ijms26104622

**Published:** 2025-05-12

**Authors:** Zhenyu Liu, Yixuan Peng, Yanshu Qu, Bin Huang, Chun Gong, Qiang Wen

**Affiliations:** 1 College of Forestry, Jiangxi Agricultural University, Nanchang 330045, China; m15396981126@163.com; 2 Jiangxi Provincial Key Laboratory of Oil-Tea Camellia Resource Cultivation and Utilization, Jiangxi Academy of Forestry, Nanchang 330032, China; 18811716268@163.com (Y.P.); ysqu@jxlky.cn (Y.Q.); huangbin007@yeah.net (B.H.)

**Keywords:** *Camellia chekiangoleosa*, fruit coloration, pigment synthesis, transcriptional regulation, *WRKY* gene family

## Abstract

The *WRKY* gene family is essential for controlling a variety of plant physiological functions, yet the involvement of specific *WRKY* members in pigment biosynthesis and accumulation in *Camellia chekiangoleosa* remains unexplored, particularly in anthocyanins and carotenoids, which play crucial roles in the pigmentation of *C. chekiangoleosa*. This study systematically identified 87 *WRKY* genes across 15 chromosomes in *C. chekiangoleosa* through bioinformatic approaches. Further structural and phylogenetic analyses of these TFs enabled their classification into six different subgroups. *WRKY* family expansion was shown to be mostly driven by tandem duplication. W-box elements, which can be binding sites for WRKY transcription factors, were present in a number of biosynthetic genes in the pigment production pathway. Yeast one-hybrid assay confirmed that five WRKY transcription factors (CchWRKY15/24/33/47/76) directly bind to the promoter regions of two key biosynthetic genes, *CchPSY1* and *Cch4CL1*. Intriguingly, among the five WRKYs tested, the expression levels of CchWRKY15, CchWRKY33, and CchWRKY47 showed the strongest positive associations with flavonoid accumulation (*p* < 0.05, Pearson correlation analysis).These findings provide novel insights into the evolutionary patterns, transcriptional regulation, and functional characteristics of CchWRKYs, while elucidating their possible regulatory mechanisms in the fruit coloration of *C. chekiangoleosa*.

## 1. Introduction

During plant growth and development, transcription factors (TFs) control the expression of downstream target genes by binding to particular DNA sequences in gene promoter regions. Basic helix–loop–helix (bHLH), myeloblastosis (MYB), Ethylene-responsive factor (ERF), No apical meristem (NAM), ATAF1/2 and CUC2 (cup-shaped cotyledon) (NAC), basic leucine Zipper (bZIP), C2H2, and WRKY are the major transcription factor families in flowering plants [1]. The *WRKY* gene family, which is one of the biggest in higher plants, is named for the conserved seven-peptide sequence, WRKYGQK [2]. WRKY TFs are defined by one or two WRKY domains, which are composed of around 60 amino acid residues in a highly conserved peptide sequence. By precisely binding to the cis-acting element W-box ((C/T)TGAC(T/C)) via the WRKY domain, WRKY transcription factors can either activate or inhibit the transcription of downstream genes [3]. They play a significant role in the growth and development of plants [4].

WRKYs (WRKY transcription factors), as key components of transcriptional regulation associated with immune responses, have been extensively studied as plant defense-related transcription factors for a long time [5,6]. The production and control of secondary metabolites have also been linked to WRKYs in recent years; some investigations suggest that these transcription factors can interact with important enzymes in the pigment manufacturing pathway [7]. OscWRKY1 can interact with the W-box elements in the promoters of *Phenylalanine ammonia-lyase* (*PAL*) and *Cinnamate 4-hydroxylase* (*C4H*), thereby influencing their expression [8]. In *Nicotiana benthamiana*, NbWRKYs can activate the expression of *C4H* and *4-coumarate CoA ligase* (*4CL*) [9]. In *Solanum lycopersicum*, SlWRKYs interact with the promoter of the pigment-related gene *Phytoene synthase* (*PSY*) and activate its expression [10].

Previous studies on WRKYs in *Camellia* plants are limited, with these studies mainly focusing on abiotic stress, squalene synthesis, etc. [11,12,13,14,15]. In studies on pigment synthesis in *Camellia* species, WRKYs is rarely mentioned [16,17,18]. *C. chekiangoleosa* is an important and special oil-producing and ornamental economic tree species in China. The seed oil from *C. chekiangoleosa* is semi-transparent with a tea-brown color. It is high in unsaturated fatty acids, rich in essential amino acids, and contains micronutrients such as zinc and selenium, making it a high-quality and health-promoting cooking oil. In production applications, red pericarp is a key indicator in the selection of superior varieties of *C. chekiangoleosa.* Therefore, exploring the mechanisms of *C. chekiangoleosa* pericarp pigment synthesis, analyzing the CchWRKYs, and revealing its regulatory mechanism have important practical significance [19]. The genome sequencing of *C. chekiangoleosa* was first completed in 2022, facilitating the exploration of its WRKYs mechanisms [20].

The main focus of this inquiry was on carotenoids and anthocyanins. Anthocyanins are an essential part of the flavonoid biosynthesis process and are crucial in defining the color of plants [21]. Fruit coloration is also greatly influenced by carotenoids, which are mostly composed of orange xanthophylls and yellow carotenes. Furthermore, carotenoids aid in photosynthesis by absorbing and distributing light energy and shielding chlorophyll from harm [22]. Important biosynthetic genes for these pigments are closely linked to fruit coloring. PSY1 mutations are affected in fruit coloring due to the interruption of the synthesis of lycopene [23]. In apples, anthocyanin accumulation and fruit colors are positively correlated with *4CL*’s expression levels in various member samples [24]. Furthermore, as byproducts of processing, natural fruit pigments have additional value. Research indicates that natural anthocyanins can be used in place of artificial red coloring in baked items and beverages. Cultivated loquats contain carotenoids that may be used to create natural colorants that vary from orange to yellow [25]. However, little is known about how WRKY transcription factors in *C. chekiangoleosa* regulate the transcription of biosynthetic genes for important pigments.

In this research, we identified the WRKYs in *C. chekiangoleosa* and systematically analyzed their chromosomal localization, subgroup classification, collinearity, gene structural characteristics, and cis-acting elements. Meanwhile, we investigated the expression levels of *WRKY*s during *C. chekiangoleosa* fruit development and focused on some important *WRKY*s that may be involved in fruit development. Further, we confirmed that members of the WRKYs may attach to the promoters of important pigment biosynthetic enzyme genes. This work provides more reliable information and guidance for studying the regulation of pigment synthesis and provides a basis for more investigation and study into the regulatory functions of WRKYs in *C. chekiangoleosa*.

## 2. Results

### 2.1. CchWRKY Protein Identification and Phylogenetic Analysis

Based on BLASTp and HMMER searches, *C. chekiangoleosa*’s reference genome revealed 87 members of the WRKYs, which were named CchWRKY01–CchWRKY87 in order according to their chromosomal locations. These proteins’ physicochemical characteristics, including their length, molecular weight, and isoelectric point, were examined. The findings demonstrated that the WRKY gene family proteins in *C. chekiangoleosa* have molecular weights ranging from 8.30 to 79.54 kDa, pI values between 4.77 and 10.05, and lengths ranging from 68 to 735 amino acids (Table S1).

**Figure 1:**
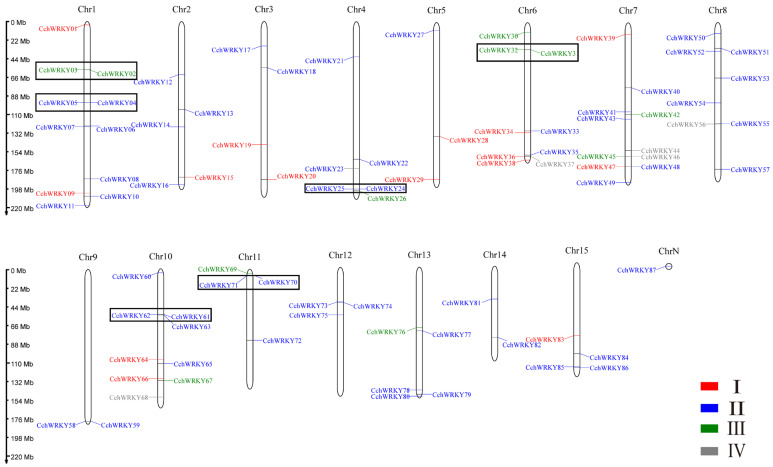
Chromosomal localization and gene clusters of *CchWRKY*s. Four colors are used to distinguish the groups corresponding to the genes. Red font represents Group I, blue font represents Group II, green font represents Group III, and gray font represents Group IV. The tandemly repeated genes are within the black boxes.

The distribution of these genes on chromosomes is shown in Figure 1. The most *WRKY* genes were found on chromosomes 1 and 7, each of which contained 11 genes, while chromosomes 9 and 14 had the fewest, each with just 2 genes. *CchWRKY87* was not mapped to any chromosome but still contained a *WRKY* domain. A total of 11 gene clusters involving 22 *CchWRKY*s were found. The *CchWRKYs* were clustered irregularly on the chromosomes, with gene clusters present on only 8 out of the 15 chromosomes. Additionally, 6 tandem duplication events were identified among these 11 gene clusters, involving 12 *CchWRKYs*.

**Figure 2:**
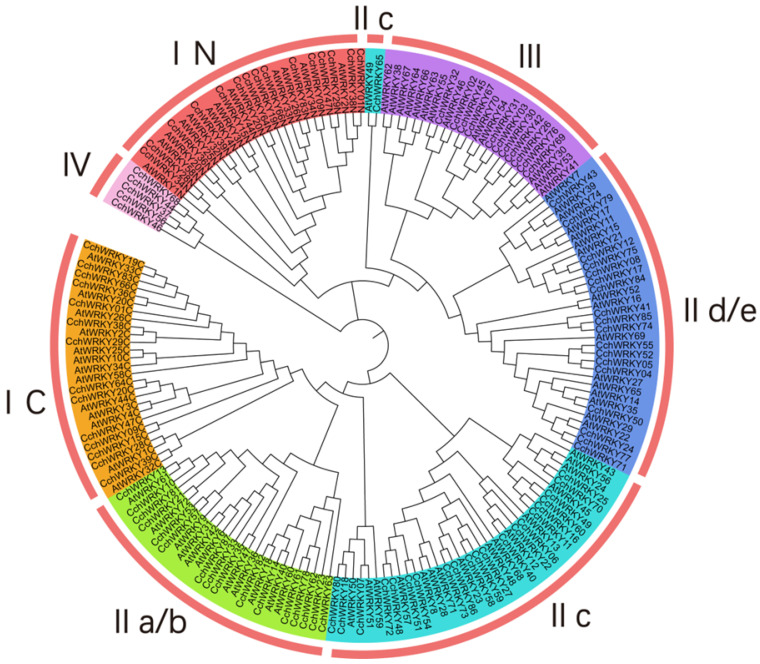
Phylogenetic tree of *WRKY* genes from *Camellia chekiangoleosa* and *Arabidopsis thaliana*. The N-terminal and C-terminal WRKY domains of Group I are represented by the groups with the letters N and C. WRKY groups I, II, III, and IV, as well as their subgroups IIa/b, IIc, and IId/e, are represented by distinct colors.

To classify the members, we performed a phylogenetic study of the conserved domain sequences of 72 *Arabidopsis thaliana* WRKY TFs (AtWRKYs) and 87 CchWRKYs by using the neighbor-joining (NJ) approach. As shown in Figure 2, there were four main groups of CchWRKYs, as follows: I, II, III, and IV. There were 15 members in Group I, 55 in Group II, 11 in Group III, and 5 in Group IV. The WRKY domains at the N- and C-terminals were used to further split Group I into two subgroups. IIa/b, IIc, and IId/e were the three subgroups that made up Group II. To make it easier to see their evolutionary branches, WRKY protein members with partially or totally deleted zinc-finger motifs were categorized into Group IV. Their distribution positions on the chromosomes are shown in Figure 1. AtWRKY49 and CchWRKY65 were assigned to Group II based on their zinc-finger motifs, while they unexpectedly formed a subgroup IIc that was closer to Group III.

### 2.2. Examination of CchWRKY Gene Structures and Protein Conserved Motifs

**Figure 3:**
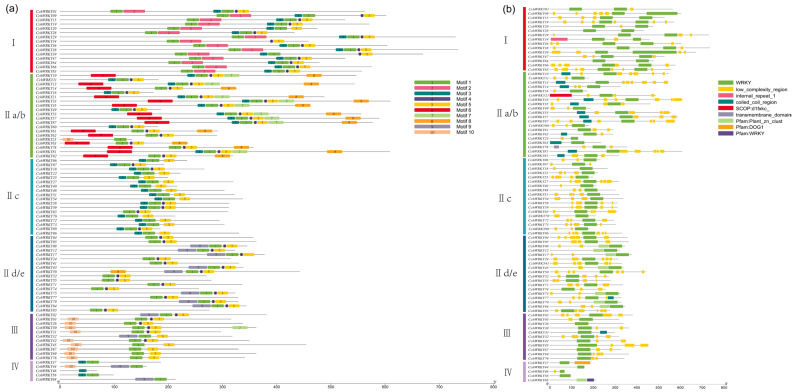
Represents motif (**a**) and conserved domain (**b**) distribution analysis of CchWRKYs.

To investigate the variety and resemblance of the CchWRKY protein motifs in more detail, we analyzed 10 conserved motifs of CchWRKY proteins (Figure 3a and Figure S1, Table S2). The findings showed that there were somewhere between one and seven motifs in the various CchWRKY proteins. The conserved motifs shared by CchWRKYs in the same group or subgroup were nearly similar. Except for CchWRKY46 in Group IV, all other members contained motif 1. In Group I, the conserved WRKY domain’s N-terminal included motif 2, whereas the C-terminal contained motif 3 (Figure 3b). Among the 87 CchWRKYs, 81 included at least one full WRKY domain, which is made up of about 60 amino acids. The WRKY domains of CchWRKY37, CchWRKY44, CchWRKY46, CchWRKY56, and CchWRKY68 were incomplete, and they all belonged to Group IV.

**Figure 4:**
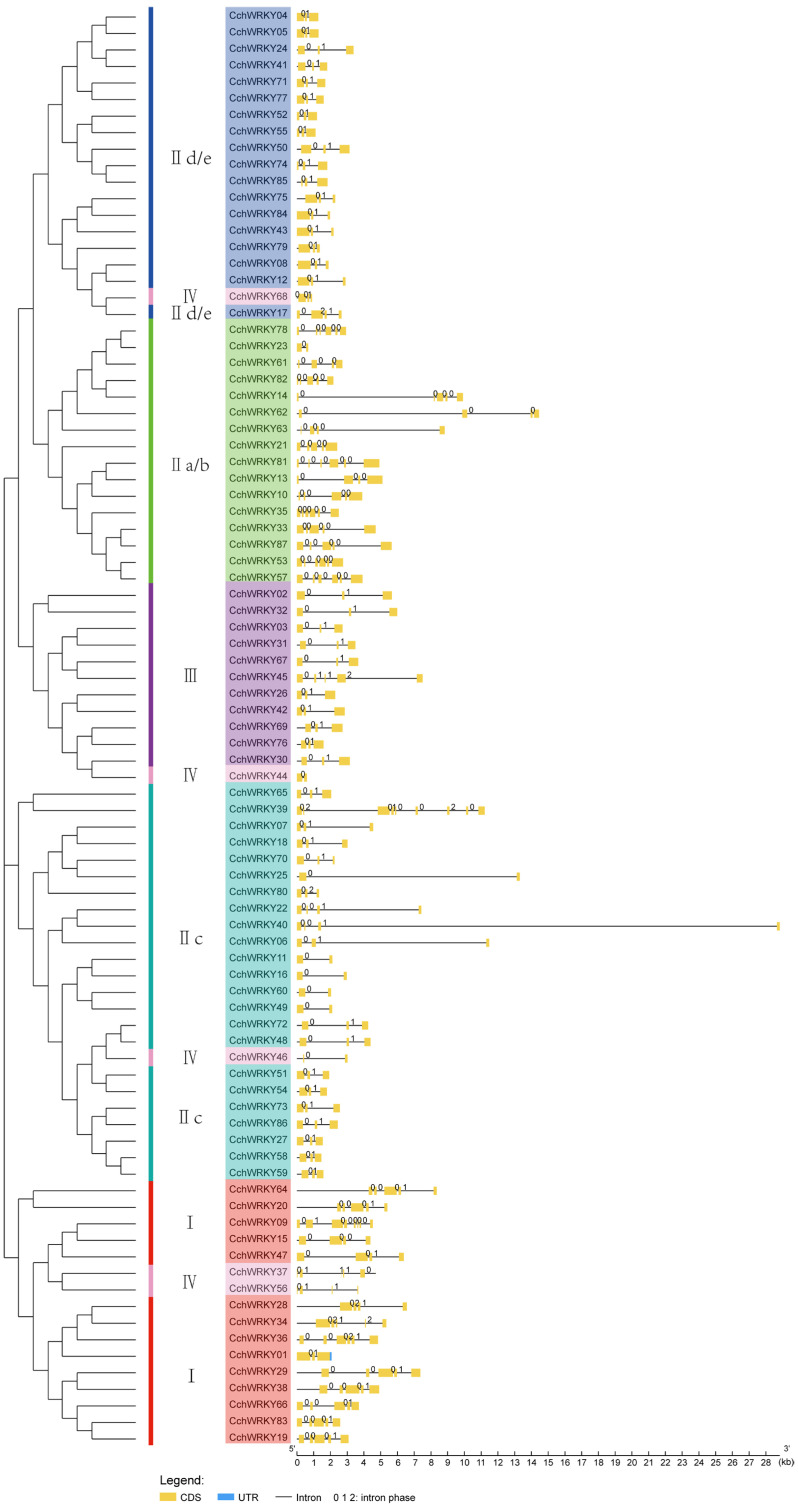
Gene structure analysis of *CchWRKY*. The coding sequence is shown by the yellow portion, introns are shown by the black short lines, the untranslated area is shown by the blue portion, and the numbers on black short lines indicate intron phases.

Apart from the WRKY domain, some family members also possessed other domains. There were distinct differences between the domains of members among different groups, while the domains of those that were grouped together were somewhat comparable (Figure 3b). Gene structure analysis revealed that most *CchWRKY* genes contained 2–4 introns, with a maximum of 8 (*CchWRKY39*) and a minimum of 1 (7 in total, mostly in Group IIc). Members of Group I generally had a larger number of introns, mostly more than four, and one member (*CchWRKY09*) had seven introns (Figure 4). In Group III, except for *CchWRKY45* with four introns, the other members had only two introns. Group II was rather unique. In subgroup IId/e, except for *CchWRKY17* with three introns, the other members had only two introns. In subgroup IIc, except for *CchWRKY39*, which had the maximum of eight introns, other members had between one and three introns. Subgroup IIa/b had noticeably more introns on average than the other two subgroups of Group II.

### 2.3. Collinearity Analysis of CchWRKYs

**Figure 5:**
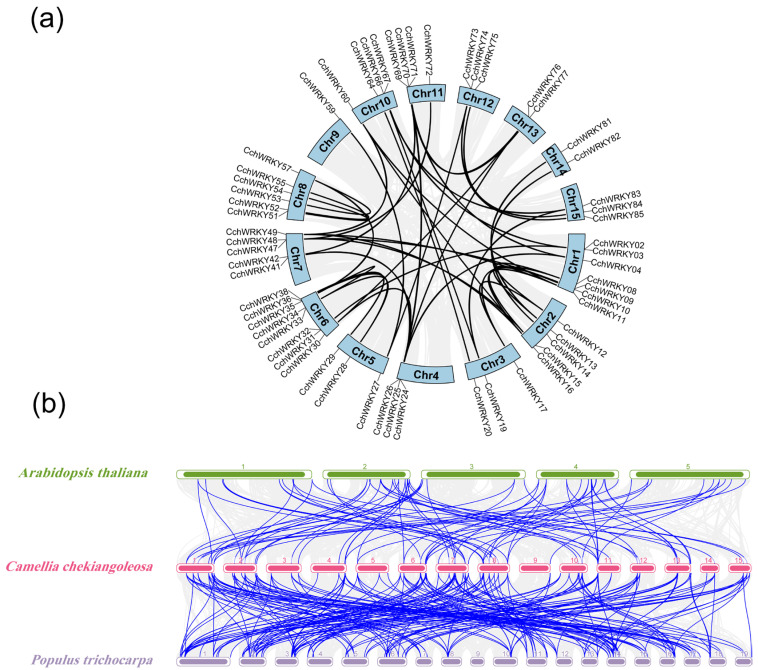
Collinearity analysis of *CchWRKY*s. (**a**) *CchWRKY* homologous gene pairs in whole genome. Black lines link the homologous gene pairs of *CchWRKY*, whereas gray lines depict the background whole genome. (**b**) *CchWRKY*s interspecies collinearity study within *Populus trichocarpa* and *A. Thaliana* genomes. Blue lines indicate collinear genes of the *WRKY* family, while gray lines indicate collinear genes in general. Numerical labels denote chromosome numbers.

A collinearity study was performed to look into the gene duplication occurrences in the *CchWRKY* family. The ka/ks and EffetiveLen of orthologous gene pairs are shown in Table S3. The data showed widespread segmental duplication during the evolution of the gene family, with 50 segmental duplication events encompassing 61 *CchWRKY* genes (Figure 5a). We also conducted a collinearity study of *WRKY* genes between *C. chekiangoleosa* and two model plants (*A. thaliana* and *Populus trichocarpa*) to explore the evolutionary patterns of the *CchWRKY* gene family and provide a framework for cross-species comparison. It showed that *CchWRKY*s exhibited a stronger collinear relationship with the WRKY family of the woody plant *P. trichocarpa* than with that of *A. thaliana*. Between *C. chekiangoleosa* and *A. thaliana*, 81 pairs of collinear *WRKY* genes (including duplicate *CchWRKY*s) were found, and 210 pairs of collinear *WRKY* genes (including duplicate *CchWRKY*s) were identified between *C. chekiangoleosa* and *P. trichocarpa* (Figure 5b).

### 2.4. Examination of Cis-Acting Components in CchWRKY Promoters

**Figure 6:**
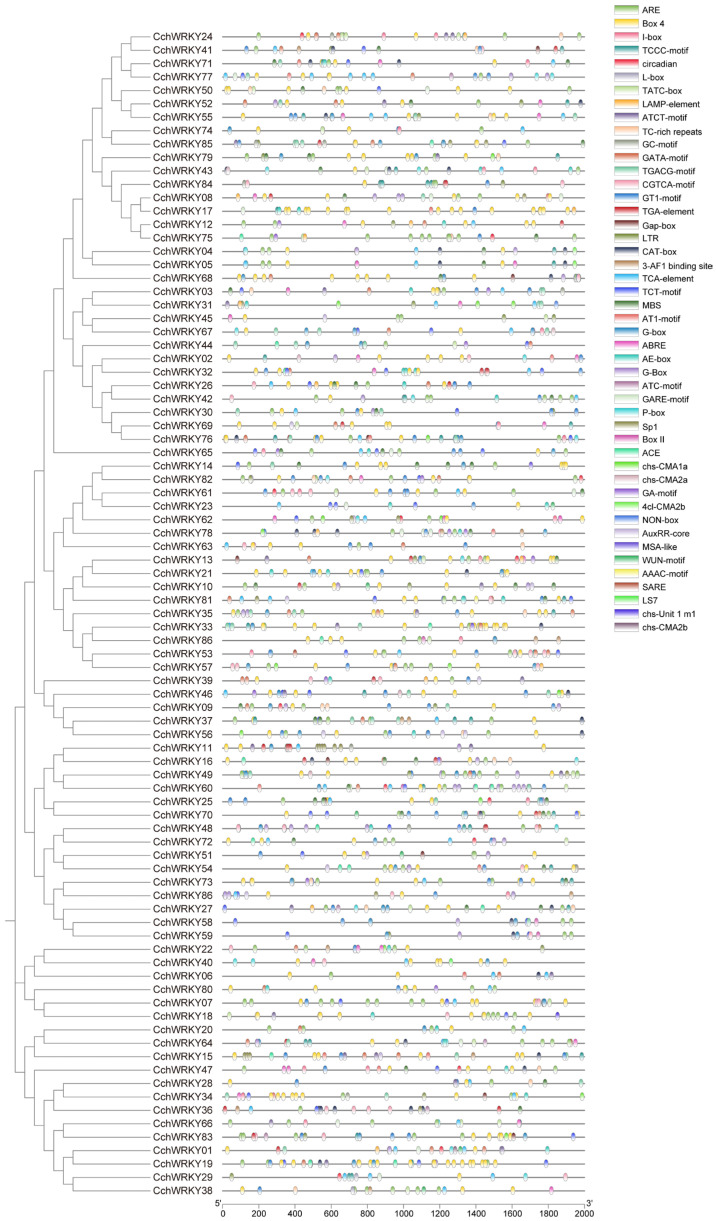
Examination of cis-acting components in CchWRKY promoters.

Bioinformatic analysis of the 2000 bp upstream promoter regions revealed that *CchWRKY*s harbor multiple stress- and hormone-responsive cis-acting elements, including light-responsive (Sp1 and GT1-motif), low-temperature-responsive (LTR), auxin-responsive (AuxRR and TGA), salicylic acid-responsive (TCA and SARE), and gibberellin-responsive elements (GARE-motif and TATC-box) (Figure 6). These elements are associated with plant growth, development, and defense responses.

### 2.5. Examining and Assessing the Expression Levels of CchWRKY Genes in Camellia chekiangoleosa Pericarp at Step Development Stages

**Figure 7:**
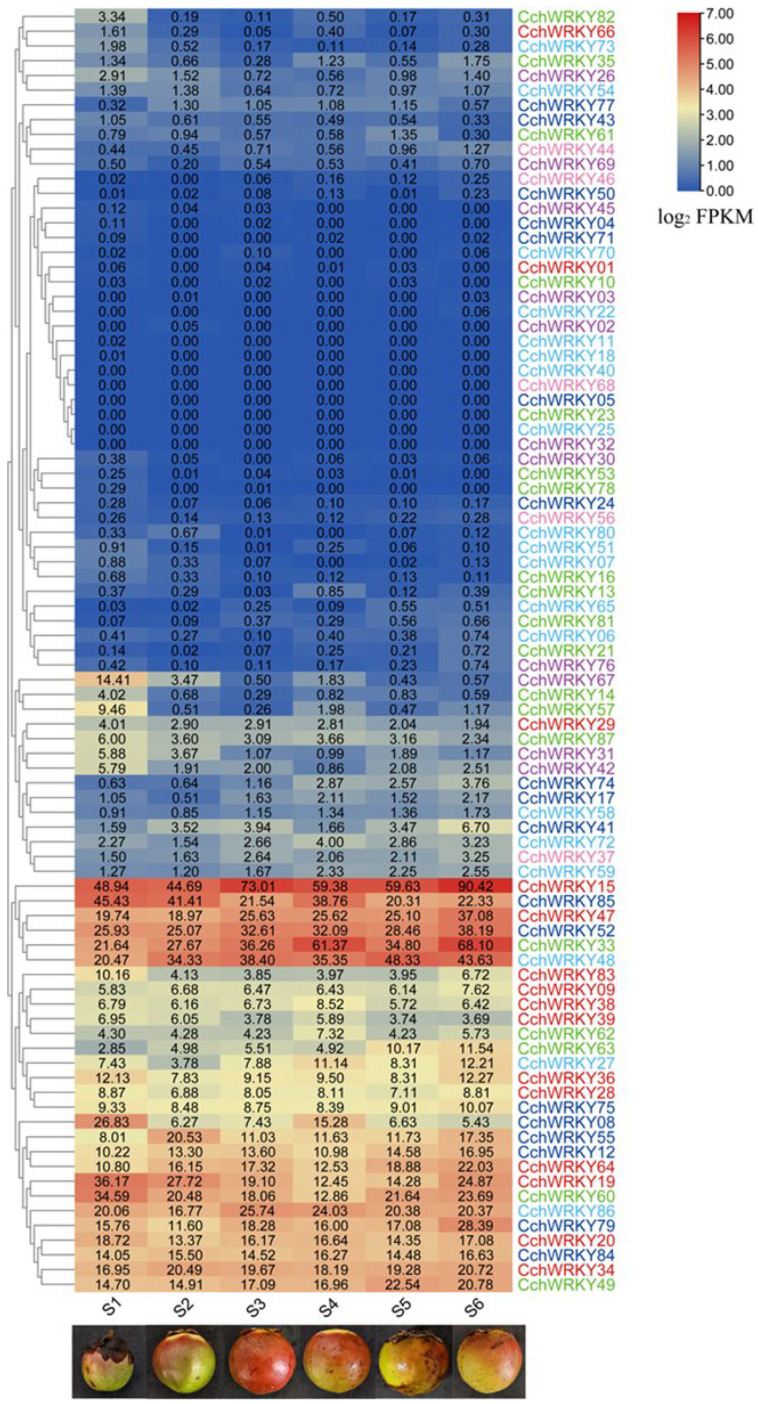
A heatmap showing the *CchWRKY* expression level in fruit samples at various phases of development. Blue denotes low expression, whereas red denotes strong expression. The six sample periods are indicated by S1–S6. Group I is represented by red, Group IIa/b by green, Group IIc by light blue, Group IId/e by dark blue, Group III by purple, and Group IV by pink color coding.

We employed RNA-seq data to measure the expression of the *WRKY* family throughout six developmental stages, from the initial fruit formation stage to the ripening stage, in order to better understand the expression levels of *CchWRKY*s during fruit development (S1–S6). As shown in Figure 7, some *CchWRKY*s were highly expressed during pericarp development, such as *CchWRKY15*, *CchWRKY47*, and *CchWRKY85*. In contrast, some *WRKY* members were not expressed at any stage of pericarp development, such as *CchWRKY25*, *CchWRKY32*, and *CchWRKY40*.

Meanwhile, a cluster analysis based on expression levels was performed on *WRKY* family members. According to the data, Group II accounted for the majority of the *CchWRKY*s that had a continuously high expression throughout the process, followed by Group I. These genes exhibited a relatively consistent high-expression trend during the entire development process. In contrast, members with low or no expression mostly belonged to Group III and Group IV. This indicates that the *CchWRKY*s in Groups I and II may be involved in important biological pathways during the pericarp development of *C. chekiangoleosa*. Additionally, this also confirms the closer evolutionary relationship between Groups I and II.

### 2.6. The Promoters of Pigment Biosynthetic Genes Could Be Directly Bound by CchWRKYs

**Figure 8:**
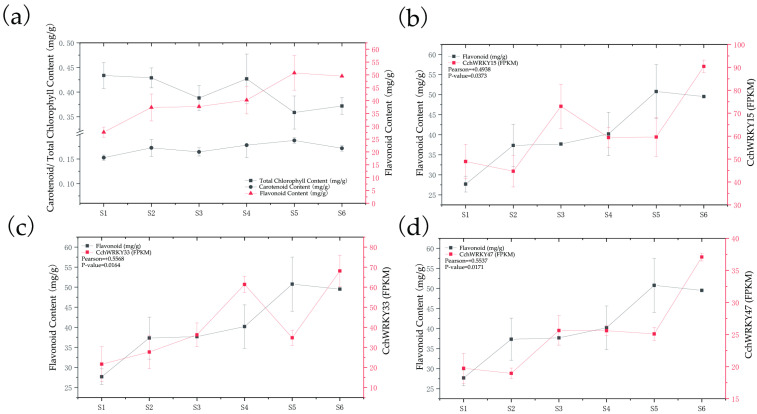
(**a**) Comparison of temporal trends in three pigment contents during different developmental stages. (**b**) Correlation between *CchWRKY15* expression levels and flavonoid content during different developmental stages. (**c**) Correlation between *CchWRKY33* expression levels and flavonoid content during different developmental stages. (**d**) Correlation between *CchWRKY47* expression levels and flavonoid content during different developmental stages.

**Figure 9:**
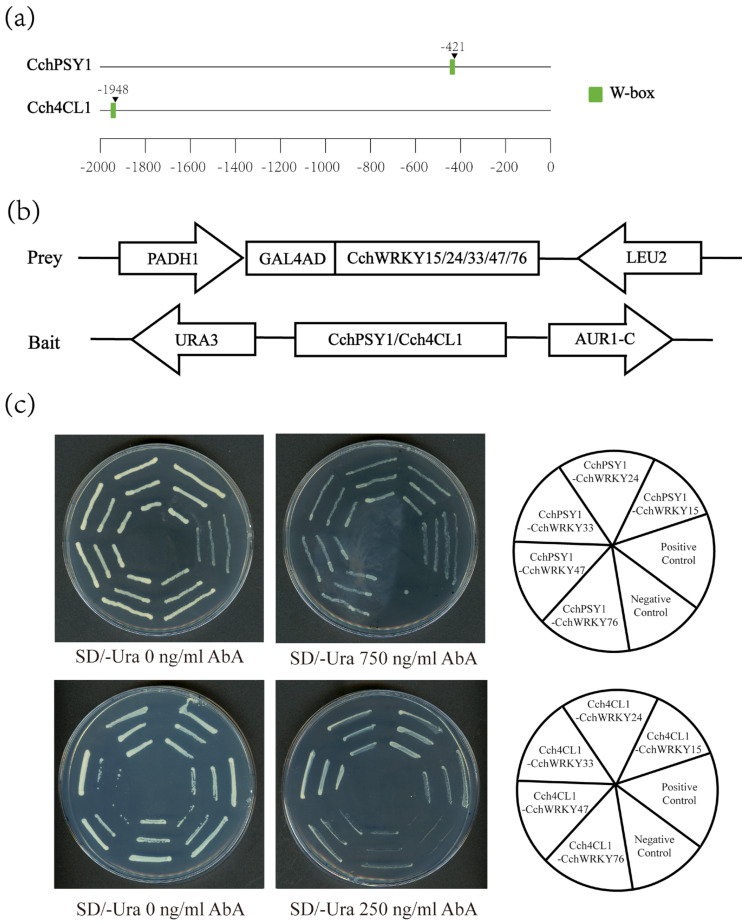
Yeast one-hybrid assay. (**a**) Positions of W-boxes in the promoter sequences of *CchPSY1* and *Cch4CL1*; (**b**) schematic diagrams of the structures of prey and bait expression vectors; and (**c**) direct binding of CchWRKYs and *CchPSY1* and *Cch4CL1* in the yeast system. The interactions between pGADT7-53 and pAbAi-53 and pAbAi-PSY1/4CL1 serve as positive and negative controls, respectively.

To elucidate the key pigments driving fruit coloration, we systematically quantified pigment content (including carotenoids, chlorophyll, and flavonoids) across six critical developmental stages. The results indicated that carotenoids and flavonoids followed similar patterns in relation to the deepening of fruit peel color, which prompted us to focus on investigating whether WRKYs exerted a potential regulatory role over these two pigments (Figure 8a). We identified the primary biosynthetic genes in the pigment synthesis pathway that were significantly expressed based on the transcriptome and genome data of *C. chekiangoleosa*. By referring to the reported *WRKY* target gene sets in other species, firstly, we screened out 5 important biosynthetic genes involved in *C. chekiangoleosa*’s pigment synthesis pathway, including *PSY*s, *4CL*s, *PAL*s, *C4H*s, and *PDS*s, as well as the following 13 candidate prey proteins from the WRKY family: CchWRKY15, CchWRKY19, CchWRKY24, CchWRKY33, CchWRKY41, CchWRKY47, CchWRKY53, CchWRKY57, CchWRKY66, CchWRKY69, CchWRKY76, CchWRKY83, and CchWRKY87. The results of the Y1H assay showed that, after transforming with *pGADT7-CchWRKY15/24/33/47/76*, the transcriptional activities of *CchPSY1* and *Cch4CL1* were significantly activated. This suggests that the promoters of *CchPSY1* and *Cch4CL1* may be directly bound by these CchWRKYs, thereby regulating pigment synthesis in *C. chekiangoleosa* (Figure 9). Pearson correlation analysis revealed that the expression levels of three *WRKY*s—*CchWRKY15*, *CchWRKY33*, and *CchWRKY47*—exhibited statistically significant positive correlations with flavonoid content (*p* < 0.05), thereby reinforcing their potential regulatory roles in pigment biosynthesis (Figure 8b–d).

## 3. Discussion

More than 1000 TFs, categorized into 58 families according to their DNA-binding domains, are found in the genomes of the majority of angiosperms [26], and the WRKY family is one of them. SPF1 (SweetPotato-Factor-1), the first member of the WRKY family, was cloned from sweet potatoes (*Ipomoea batatas* (L.) Lam.) in 1994 [27]. Thanks to developments in sequencing technology and bioinformatics, *WRKY* genes have since been found and examined in more and more plant genomes, including *A. thaliana*, *Zea mays* L., *Citrullus lanatus*, and grapes [28,29,30,31]. Multiple studies related to the WRKY family have been performed in *Camellia* plants and model plants. Functional characterization has revealed that *CsWRKY* genes in tea plants are responsive to both heat and cold stresses [32]. Light-regulated CoWRKY may contribute to increases in squalene content in *C. oleifera* seeds [15]. Salicylic acid treatment caused 15 CjWRKYs to be expressed in *Camellia japonica* [33]. AtWRKY40 and AtWRKY63 regulate stress-responsive genes that encode mitochondrial and chloroplastic proteins in *A. thaliana* [34]. Studies on WRKY cover a wide range of crops and fields. The link between CchWRKY and natural pigments in *C. chekiangoleos* is the main subject of this work, which improves our knowledge of *WRKY*’s function in controlling plant development and provides fresh insight into the roles of *WRKY* genes in the regulation of pigment synthesis.

In total, 87 members of the *WRKY* gene family (CchWRKY1 through to CchWRKY87) were finally found in *C. chekiangoleosa* (Figure 1). Nevertheless, these genes were not evenly distributed throughout chromosomes. Related species have been found to have comparable numbers of *WRKY* genes, such as 80 in *C. Sinensis* and 89 in *C. oleifera* Abel [35,36]. Meanwhile, this number is higher than that in *Ginkgo biloba* (40 genes), but lower than that in *Arachis hypogaea* (an allotetraploid, 158 genes) and *Fragaria ananassa* (an octoploid, 222 genes) [37,38,39]. These species’ genome sizes differ (*C. chekiangoleosa*, 2.73 Gb; *C. sinensis*, 3.26 Gb; *C. oleifera*, 2.95 Gb; *G. biloba*, 9.87 Gb; *A. hypogaea*, 2.7 Gb; and *F. ananassa*, 268 Mb). In general, genome size is directly correlated with the number of *WRKY* family members. However, in certain species, the number of WRKY genes appears to be independent of genome size, maybe as a result of intricate historical processes (such as genome duplication). More research is still required to determine the precise causes of this.

A commonly used categorization method for the *WRKY* gene family was developed in 2000 based on the traits of the *WRKY* gene family in *A. thaliana*. It separates genes into three groups (I, II, and III) according to the quantity of WRKY domains and the properties of zinc finger-like motifs [40]. On the basis of this classification, this study referred to the methods used in relevant research on willow, tea, and mango [32,41,42]. Phylogenetic tree analysis indicated that the C-terminal WRKY domain of WRKY TFs primarily mediates DNA-binding activity, whereas the N-terminal WRKY domain may serve as an interface for protein–protein interactions or as an auxiliary function in the binding process (zinc fingers stick together). Similar to some studies, a small number of WRKY proteins do not conform to the characteristics of these three groups. They still have one WRKYGQK motif even if they exhibit the partial or whole deletion of the zinc-finger motif [43,44]. For instance, AtWRKY10 has a single WRKY domain, possibly due to the loss of the N-terminal WRKY domain, yet its structure is consistent with Group I’s characteristics [45]. Referring to the classification method in apple-related studies, this type of WRKY member was classified as Group IV [46]. From the phylogenetic tree in this paper, it can be seen that Group IV clustered with the N-terminal of Group I. Consequently, we deduce that several members of WRKY Group I lost their C-terminal zinc-finger domains throughout the evolutionary process of *C. chekiangoleosa*, suggesting that gene mutations may have taken place at this time. In addition, the heptapeptide domains of three members (CchWRKY07, CchWRKY18, and CchWRKY80) in subgroup IIc were mutated to WRKYGKK. The mutation of the heptapeptide structure to WRKYGKK in WRKY transcription factors would cause WRKY to bind to other cis-acting elements instead of the normal W-box that binds to WRKYGQK [47]. The variety of these distinctive motifs offers a new foundation for investigating the roles and development of these CchWRKY family members.

One of the main forces behind genome evolution is gene duplication occurrences. Many tandem and segmental duplication events frequently accompany the development of plant genomes, which are among the mechanisms driving gene amplification and functional diversification [48]. Numerous homologous gene pairs suggested that *CchWRKY* underwent substantial segmental duplication events (Figure 5). The uneven chromosomal distribution of *CchWRKY*s suggests that segmental duplication may drive their family expansion in *C. chekiangoleosa*. The collinearity study results showed that 59 *CchWRKY*s were collinear with the *WRKY* genes in both *A. thaliana* and *P. trichocarpa*, indicating that these genes likely existed before species divergence and were highly conserved. This might offer hints for more research on the *WRKY* gene family’s evolution.

The vibrant fruit peel color is one of the distinctive features of *C. chekiangoleosa* that sets it apart from other species in the *Camellia* genus. It is worth noting that the promoters of *CchWRKY*s contain multiple light- and temperature-responsive elements. Since light and temperature are the main environmental factors affecting pigment synthesis in plants, we hypothesize that *CchWRKY*s may play a role in controlling *C. chekiangoleosa*’s pigment content [49]. Research has demonstrated a strong correlation between the differential expression of pigment-related genes and WRKY transcription factors [50,51]. The fruit color of economic crops is a comprehensive manifestation of pigments such as carotenoids and flavonoids, which act as secondary metabolites in plants and participate in their growth and development processes [52,53]. Key biosynthetic genes (e.g., *PAL/CHS/CHI/F3H* in anthocyanin synthesis and *PSY/PDS/LCYb* in carotenoid production) are central to plant pigment metabolism through transcription-factor-mediated regulation. Hossain et al. suggested that *4CL* and *CHS* are controlled by transcription factors like FaMYB5 in strawberry [54]. MabHLH3 can regulate the accumulation of anthocyanins in mulberries by activating the expression of important genes, including *CHS*, *CHI*, *F3H*, *DFR*, and *UFGT* [55]. The transcription of genes linked to carotenoid synthesis, including *CCS*, *PSY*, and *β-CH1*, is activated in pepper by the MYB transcription factor DIVARICATA1 [56]. Combining our Y1H results, the synthesis and accumulation of pigments in *C. chekiangoleosa* are probably regulated by the transcriptional activation of biosynthetic genes, with CchWRKYs acting as key transcriptional modulators.

Currently, there is no research on the functions of CchWRKY. Referring to the previous method exploring WRKY TFs in *Salix suchowensis* [40], we mined potential WRKY-targeted binding gene sets in pigment synthesis [57,58]. We discovered five CchWRKYs (CchWRKY15/24/33/47/76) as new regulators of *CchPSY1* (carotenoid pathway) and *Cch4CL1* (flavonoid pathway) by Y1H analysis. The statistically significant correlations between the expression levels of *CchWRKY15*, *CchWRKY33*, and *CchWRKY47* and flavonoid accumulation across developmental stages (Figure 8b–d) strongly suggest that they play specialized roles in regulating this pathway. While our Y1H assays detected their binding to both *CchPSY1* (carotenoid-related) and *Cch4CL1* (flavonoid-related) promoters (Figure 9), the temporal coupling of their expression with flavonoid dynamics—but not carotenoid levels—implies that *Cch4CL1* may be their primary functional target. This divergence between in vitro binding capacity and in vivo metabolic coordination highlights the importance of spatiotemporal context in transcriptional regulation. Future studies should prioritize validating these WRKYs as flavonoid-specific regulators through EMSA and *4CL1* promoter mutagenesis, while exploring why their putative *PSY1* binding fails to translate into carotenoid correlation—possibly due to post-translational modifications or competing regulatory inputs. For breeding and industrial applications, high-expression candidate *CchWRKY*s can serve as genetic markers to facilitate the selection of superior fruit varieties. At the same time, these natural pigments hold significant industrial value, with strong potential for large-scale production in the food, cosmetics, and dietary supplement industries.

Nevertheless, there is a need for more experiments incorporating techniques such as ChIP-seq to confirm regulatory universality. The mechanistic interplay between environmental cues (particularly light and thermal signals) and the WRKY-mediated transcriptional regulatory networks governing pigment biosynthesis warrants in-depth exploration to elucidate the spatiotemporal control mechanisms involved.

## 4. Materials and Methods

### 4.1. Fruit Materials of C. chekiangoleosa

At the Linfeng Forestry Farm (28°57′ N, 115°39′ E) in Yongxiu County, Jiujiang City, Jiangxi Province, samples of *C. chekiangoleosa* of the same clone were gathered. From 22 June to 1 September 2023, fruits were collected every 14 days, yielding six sampling events (designated as S1–S6). The initial sampling (S1) coincided with the early seed kernel formation phase of *C. chekiangoleosa*, while the final sampling (S6) occurred during the maturation phase, characterized by natural dehiscence and seed shedding in a subset of fruits. In order to prepare it for DNA/RNA extraction, the fruit pericarp was immediately removed, frozen in liquid nitrogen, and then kept in a refrigerator at −80 °C.

### 4.2. Genome-Wide Identification of CchWRKYs

The genomic data of *C. chekiangoleosa* were published by our research team in 2022 and are available for open access at https://ngdc.cncb.ac.cn/gwh/ (accessed on 9 April 2024) [20]. The Pfam database provided the Hidden Markov Model (HMM) file for the WRKY domain (PF03106) (http://pfam.xfam.org/, accessed on 12 April 2024) [59]. Members of the WRKY gene family were sought out in *C. chekiangoleosa* using HMMER 3.0, yielding preliminary candidate members of the *CchWRKY* gene family [60]. Subsequently, the protein sequences of *C. chekiangoleosa* were searched using the BLASTp program (version 2.15.0+), with the WRKY of *Arabidopsis thaliana* (obtained from TAIR10, www.arabidopsis.org, accessed on 13 April 2024) as query sequences (E-value = 1 × 10^−3^, minimum sequence identity = 80%) [61,62]. The results were cross-verified with those from the previous step to obtain potential members of the *CchWRKY* gene family [41].

Using the SMART website (http://smart.embl.de, accessed on 15 April 2024), the acquired sequences were first categorized and confirmed to be in the WRKY domain [63]. Expasy, an online tool (http://web.expasy.org/compute_pi/, accessed on 20 April 2024), was used to examine the molecular weights (Mw value) and isoelectric points (pI value) of all WRKY proteins [64]. Protein length was calculated using the TBtools-II software (version v2.210) [65].

### 4.3. Chromosomal Localization, Multiple Sequence Alignment, and Phylogenetic Analysis of CchWRKYs

The online program MG2C (http://mg2c.iask.in/mg2c_v2.0/, accessed on 3 May 2024) was used to display the chromosomal location data of *CchWRKY*s [66], which were acquired using the TBtool-II software. Muscle (version 5.3) was used to align the CchWRKY proteins’ full-length sequences [67]. After the WRKY domains from *C. chekiangoleosa* and *A. thaliana* were aligned numerous times, including the N-terminal and C-terminal domains, a phylogenetic tree was created in MEGA 11 using the neighbor-joining (NJ) technique, with bootstrap set to 1000. Lastly, the online tool ITOL (http://itol.embl.de, accessed on 15 May 2024) was used to show the phylogenetic tree.

### 4.4. Analysis of Gene Structure, Conserved Motifs, and Conserved Domains of CchWRKYs

Using TBtools-II, annotation data for every *CchWRKY* gene were taken out of the genome annotation file for *C. chekiangoleosa*. Subsequently, exon–intron structure diagrams were obtained using the online tool GSDS (Gene Structure Display server, http://gsds.cbi.pku.edu.cn/, accessed on 17 May 2024) [68]. The MEME website was used to forecast and examine the conserved motifs (https://meme-suite.org/meme/tools/meme, accessed on 18 May 2024), with the maximum number of motifs set to 10 [69]. The visualization of the conserved domains of CchWRKYs was completed using the Batch SMART module in TBtools-II.

### 4.5. Analysis of Cis-Acting Elements in the Promoter Region and Collinearity of CchWRKYs

The interspecies collinearity study was performed using the MCScan X module of TBtools-II, and the TBtools-II software was utilized to show the findings. The PlantCARE website was used to extract the sequences 2000 bp upstream of the 5′ ends of *CchWRKY*s and anticipate the cis-acting components (http://bioinformatics.psb.ugent.be/webtools/plantcare/html/, accessed on 18 May 2024) [70]. For visualization, the TBtools-II software’s Simple Bio Sequence Viewer tool was utilized.

### 4.6. Analysis of CchWRKYs’ Gene Expression Profiles

The BWA software (version 0.7.17-r1188) (with a mismatch of ≤ 2 bp and other parameters set as default) [71] was used to map the RNA-Seq reads of the fruit samples from six growth stages to the *CchWRKY* sequences, respectively, and the number of reads mapped to each *CchWRKY* was calculated. Fragments Per Kilobase of transcript per Million fragments mapped (FPKMs) were manually computed and log2-normalized [72]. The TBtools-II software’s HeatMap Illustrator plugin was used to create a heatmap of the gene expression profiles. The expression levels of biosynthetic genes for important enzymes in the pigment manufacturing pathway of the *C. chekiangoleosa* samples across six phases are supplemented in Table S4 (with three replicates per stage).

### 4.7. DNA/RNA Extraction and Cloning of CchWRKY Members

RNA was extracted using the TSINGKE TSP0201 Trelief Hi-Pure Plant RNA Kit (Tsingke Biotechnology Co., Beijing, China), and DNA was extracted with the Hi-Pure Plant Genomic DNA Kit (Tsingke Biotechnology Co., Beijing, China). The cloning of *CchWRKY* was performed using the Takara EX Premier DNA Polymerase (Takara, Beijing, China). PCR amplification quality was assessed via 1.5% agarose gel electrophoresis. The Trelief DNA Cel Extraction Kit (Tsingke Biotechnology Co., Beijing, China) was used to purify PCR products for subsequent Y1H experiments.

### 4.8. Determination of Total Chlorophyll, Flavonoids, and Carotenoids

The contents of total chlorophyll and carotenoids were determined using the Plant Chlorophyll Content Assay Kit and Plant Carotenoids Content Assay Kit (BOXBIO, Beijing, China) based on spectrophotometric analysis. The contents of flavonoids were determined using the Plant Flavonoids Content Assay Kit (BOXBIO, Beijing, China).

### 4.9. Yeast One-Hybrid Assay

The Supplementary Tables S5 and S6 contain a list of the primers used for gene cloning. The NEBuilder HiFi DNA Assembly (NEW ENGLAND BioLabs, Beijing, China) kit was used for vector digestion, with pGADT7 digested using EcoR1 and BamH1, and pAbAi digested using Sac1 and Xho1. The ClonExpress II One Step Cloning Kit (Takara, Beijing, China) was used to clone *CchWRKYs* into the pGADT7 vector, named AD-prey, and the promoter regions 2000 bp upstream of the transcription start sites of *CchPSY* and *Cch4CL* were cloned into the pAbAi vector, named AbAi-bait. The constructed AbAi-bait plasmid was then digested with BstbI to linearize it for integration into the yeast chromosome. The linearized AbAi-bait plasmids were transformed into Y1HGold competent cells, which were then spread on a synthetic defined medium without uracil (SD/-Ura). Using the OD of the bacterial suspension at 0.02 as the standard, the AbA concentration that suppressed self-activation was determined. The constructed AD-prey plasmids were, respectively, transformed into the prepared competent cells. Yeast transformants were cultured on both SD/-Ura and SD/-Ura + AbA (Aureobasidin A) selection media. Yeast colonies were subjected to a 48–72 h incubation period at 30 °C, after which growth phenotypes were systematically assessed.

## 5. Conclusions

In this study, we conducted the first comprehensive analysis of 87 CchWRKYs based on the whole-genome data of *C. chekiangoleosa*, including chromosomal localization, gene structure, and evolutionary relationships. Our findings revealed that the expansion of this gene family was likely driven by segmental duplication events. Through Y1H assays, we identified the interaction between two pigment biosynthetic genes and five CchWRKYs, demonstrating their probable involvement in the regulation of pigment metabolism. Furthermore, integrating metabolite profiling with transcriptional data, we identified three WRKY transcription factors (CchWRKY15/33/47) as top candidates for flavonoid regulation. Additional validation is needed for this regulatory mechanism. These findings enhance our understanding of the composition and functional dynamics of CchWRKYs, providing new insights into the mechanisms of fruit coloration and the potential exploitation of pigment-related byproducts in *C. chekiangoleosa*. Furthermore, the potential regulation of pigment biosynthesis by environmental signals (e.g., light, temperature, and hormones) through CchWRKYs warrants focused investigation.

## Data Availability

Data will be made available on request.

## Supplementary Materials

The following supporting information can be downloaded at: https://www.mdpi.com/article/10.3390/ijms26104622/s1.
