# Supplementary material for: Genome-Wide Characterization of WRKY Gene Family in Camellia chekiangoleosa Identifies Potential Regulatory Components in Pigment Biosynthesis Pathways
Source: Int J Mol Sci. 2025 May 12;26(10):4622. doi: 10.3390/ijms26104622 (PMC12111399; doi:10.3390/ijms26104622)
Supplement: Supplementary file 1 [file ijms-26-04622-s001.zip › ijms-3502116-supplementary.pdf]

Supplementary Materials

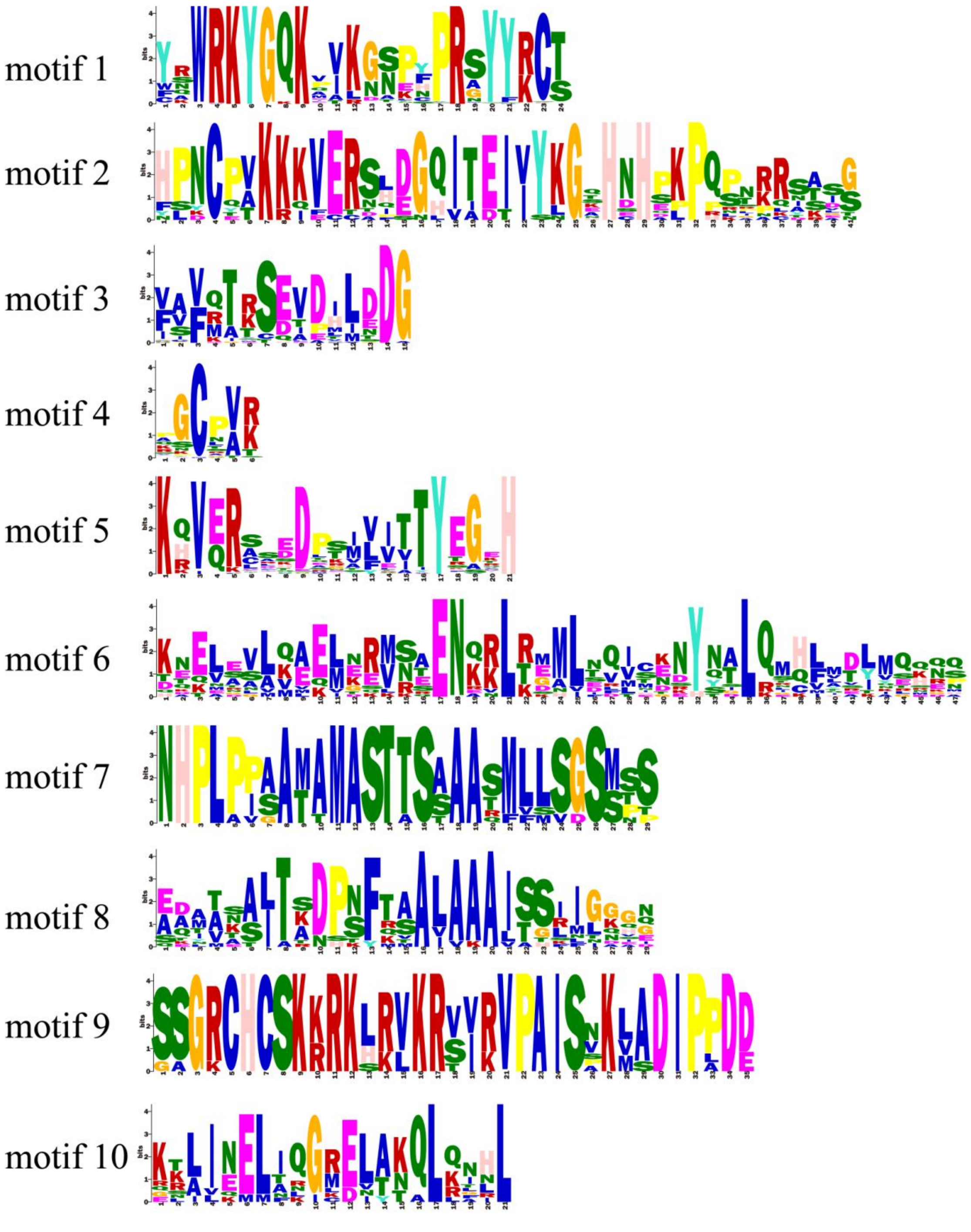

Figure S1. Motif sequence logo of *CchWRKYs*. The horizontal axis represents the base number, a nd the vertical axis represents the corrected score. The higher the base height, the higher the po ssibility of that base appearing at that position in the motif.

Table S1. Basic Information of CchWRKYs.

ID	Group	Number of Amino Acid	Molecular Weight	Theoretical pI	Instability Index	Aliphatic Index	Grand Average of Hydropathicity
CchWRKY01	I	563	61560.63	8.06	54.26	58.33	-0.66
CchWRKY02	III	381	40343.61	5.93	58.65	57.14	-0.619
CchWRKY03	III	316	34880.25	5.04	68.88	66.01	-0.529
CchWRKY04	II d/e	356	39497	7.01	62.89	55.37	-0.885
CchWRKY05	II d/e	362	40184.7	7.01	64.23	53.92	-0.898
CchWRKY06	II c	234	26572.1	9.28	60.84	52.09	-0.79
CchWRKY07	II c	192	21766.9	6.59	45.73	44.06	-1.043
CchWRKY08	II d/e	345	38146.24	9.65	50.87	74.61	-0.585
CchWRKY09	I	602	66304.88	8.78	55.27	71.71	-0.464
CchWRKY10	II a/b	547	60160.05	6.35	56.42	55.5	-0.885
CchWRKY11	II c	182	20738.4	9.54	52.06	53.46	-0.827
CchWRKY12	II d/e	322	35251.05	9.47	51.31	75.12	-0.456
CchWRKY13	II a/b	544	58736.92	7.03	44.45	59.19	-0.665
CchWRKY14	II a/b	326	36166.64	8.68	43.76	68.16	-0.698
CchWRKY15	I	526	57969.59	7.66	57.07	63.16	-0.744
CchWRKY16	II a/b	173	19782.28	9.56	52.03	50	-0.769
CchWRKY17	II d/e	377	42236.01	9.53	46.6	63.1	-0.692
CchWRKY18	II c	266	30647.92	9.16	53.77	67.07	-0.541
CchWRKY19	I	571	63260.97	6.88	62.64	45.59	-0.975
CchWRKY20	I	475	52445.13	8.5	44.84	58.46	-0.929
CchWRKY21	II a/b	488	53255.96	5.93	47.97	61.62	-0.824
CchWRKY22	II c	222	25423.78	6.5	55.33	46.04	-1.083
CchWRKY23	II a/b	133	14964.76	9.19	45.27	58.57	-0.95
CchWRKY24	II d/e	315	34796.15	5.76	52.61	45.24	-0.833
CchWRKY25	II c	199	22486.56	9.56	47.88	66.63	-0.839
CchWRKY26	III	337	37563.98	5	46.81	74.33	-0.459
CchWRKY27	II c	319	35574.73	6.83	66.39	47.96	-0.866
CchWRKY28	I	433	48368.47	8.92	47.05	58.48	-1.134
CchWRKY29	I	730	78879.7	6.01	59.62	51.59	-0.831
CchWRKY30	III	362	39743.73	5.3	56.45	53.67	-0.802
CchWRKY31	III	297	33498.29	5.08	61.08	71.52	-0.589
CchWRKY32	III	318	35137.3	6.55	54.41	58.62	-0.562
CchWRKY33	II a/b	610	66416.62	6.06	48.18	60.85	-0.73
CchWRKY34	IV	459	50431.15	5.68	52.08	56.86	-1.02
CchWRKY35	II a/b	463	51294.57	7.58	53.63	62.61	-0.635
CchWRKY36	I	604	65787.65	6.35	44.77	62.75	-0.696
CchWRKY37	IV	186	21816.19	9.78	56.37	88.98	-0.527
CchWRKY38	I	735	79538.64	6.02	52.63	54.68	-0.782
CchWRKY39	I	670	73935.79	5.77	56.9	70.72	-0.61
CchWRKY40	II c	216	24661.17	7.64	53.94	43.24	-1.037
CchWRKY41	II d/e	330	36026.79	5.92	58.99	49.36	-0.772

CchWRKY42	Ⅲ	349	39487.77	4.91	46.83	60.11	-0.715
CchWRKY43	Ⅱ d/e	338	38130.48	9.87	54.57	64.85	-0.717
CchWRKY44	Ⅳ	160	18397.81	7.8	44.09	71.31	-0.789
CchWRKY45	Ⅲ	454	50509.6	8.59	59.11	63.59	-0.788
CchWRKY46	Ⅳ	68	8300.52	10.05	50.1	48.68	-1.435
CchWRKY47	Ⅰ	526	57386.33	8.13	58.77	52.38	-0.879
CchWRKY48	Ⅱ c	303	33428.61	5.4	53.4	51.85	-0.858
CchWRKY49	Ⅱ a/b	186	21097.56	9.28	37.38	52.37	-0.854
CchWRKY50	Ⅱ d/e	442	48207.2	5.17	53.89	57.13	-0.685
CchWRKY51	Ⅱ c	322	35681.65	6.35	60.83	48.73	-0.757
CchWRKY52	Ⅱ d/e	270	29788.85	5.56	70.43	57.44	-0.809
CchWRKY53	Ⅱ a/b	556	60423.4	8.15	45.93	60.59	-0.638
CchWRKY54	Ⅱ c	337	37144.96	6.2	61.49	44.21	-0.818
CchWRKY55	Ⅱ d/e	279	31286.59	5.87	51.04	53.44	-0.854
CchWRKY56	Ⅳ	99	11416.33	9.2	49.38	89.39	-0.451
CchWRKY57	Ⅱ a/b	589	63840.05	6.53	51.33	59.05	-0.676
CchWRKY58	Ⅱ c	312	34421.33	6.01	57.2	53.43	-0.694
CchWRKY59	Ⅱ c	311	34348.28	6.01	57.35	54.21	-0.686
CchWRKY60	Ⅱ a/b	181	20584.03	9.4	37.54	53.81	-0.855
CchWRKY61	Ⅱ a/b	292	32415.13	6.66	56.15	75.48	-0.709
CchWRKY62	Ⅱ a/b	290	32655.81	6.71	36.15	67.45	-0.643
CchWRKY63	Ⅱ a/b	237	26788.5	8.25	39.78	71.52	-0.597
CchWRKY64	Ⅰ	479	52065.69	8.87	42.21	53.11	-0.904
CchWRKY65	Ⅱ c	309	34374.35	5.11	56.36	62.17	-0.638
CchWRKY66	Ⅰ	576	63992.82	8.12	60.37	40.5	-1.007
CchWRKY67	Ⅲ	329	36538.61	6.15	36.62	60.49	-0.725
CchWRKY68	Ⅳ	214	24192.71	9.85	42.33	65.09	-0.654
CchWRKY69	Ⅲ	362	40654.74	4.97	55.57	54.42	-0.767
CchWRKY70	Ⅱ c	212	23918.89	9.26	48.3	64.34	-0.897
CchWRKY71	Ⅱ d/e	336	36809.74	5.08	61.85	57.74	-0.606
CchWRKY72	Ⅱ c	294	32123.31	5.94	56.84	50.75	-0.841
CchWRKY73	Ⅱ c	303	34050.32	6.46	60.1	55.41	-0.707
CchWRKY74	Ⅱ d/e	256	29160.88	5.13	81.04	51.41	-1.118
CchWRKY75	Ⅱ d/e	323	36119.87	9.75	51.82	63.41	-0.778
CchWRKY76	Ⅲ	341	38621.78	5.45	47.44	58.3	-0.816
CchWRKY77	Ⅱ d/e	328	37129.24	5.49	62.39	61.62	-0.682
CchWRKY78	Ⅱ a/b	357	39411.45	6.2	51.91	69.92	-0.572
CchWRKY79	Ⅱ d/e	329	35898.65	9.7	48.41	62.25	-0.643
CchWRKY80	Ⅱ c	185	20567.6	5.76	44.31	45.41	-0.917
CchWRKY81	Ⅱ a/b	609	66483.79	8.36	55.86	54.98	-0.798
CchWRKY82	Ⅱ a/b	320	34981.18	8.81	45.43	67.38	-0.661
CchWRKY83	Ⅰ	557	61574.38	8.44	69.03	49.39	-0.914
CchWRKY84	Ⅱ d/e	344	38848.77	9.86	47.44	65.17	-0.853
CchWRKY85	Ⅱ d/e	275	31056.88	4.77	70.9	50.69	-1.008

CchWRKY86	II c	330	36567.05	7.68	45.17	66.73	-0.526
CchWRKY87	II a/b	577	62941.28	5.96	41.71	56.07	-0.785

Table S2. E-value, sites and width of CchWRKYs Motif sequence.

Name	E-value	Sites	Width
Motif1	5.1e-1866	94	24
Motif2	1.6e-940	81	21
Motif3	1.9e-286	15	41
Motif4	8.2e-264	42	15
Motif5	2.2e-183	15	47
Motif6	8.4e-147	8	35
Motif7	5.4e-112	9	29
Motif8	6.7e-112	14	29
Motif9	1.3e-087	80	6
Motif10	4.3e-058	11	21

Table S3. The Ka/Ks and EffetiveLen of orthologous gene pairs in the collinearity analysis of *C. chekiangoleosa*.

Seq_1	Seq_2	Ka	Ks	Ka/Ks	EffectiveLen
CchWRKY02	CchWRKY32	0.235831252	0.773073088	0.305056864	915
CchWRKY03	CchWRKY67	0.404578477	1.95474454	0.206972558	831
CchWRKY03	CchWRKY31	0.25908943	0.459752481	0.563541125	855
CchWRKY04	CchWRKY71	0.517814984	NaN	NaN	900
CchWRKY04	CchWRKY24	0.461504906	2.110508249	0.218670032	858
CchWRKY08	CchWRKY12	0.119046496	0.896487596	0.132792129	960
CchWRKY08	CchWRKY17	0.197702295	1.315701645	0.150263774	936
CchWRKY09	CchWRKY15	0.145200797	0.361353918	0.401824333	1380
CchWRKY09	CchWRKY47	0.290549143	1.066487926	0.272435473	1347
CchWRKY10	CchWRKY13	0.33585832	1.037942616	0.323580817	1413
CchWRKY11	CchWRKY60	0.244124878	1.13604417	0.214890305	528
CchWRKY11	CchWRKY16	0.136110651	0.487438835	0.27923637	507
CchWRKY11	CchWRKY49	0.260268082	1.268103185	0.205242038	534
CchWRKY12	CchWRKY17	0.209369211	1.311464982	0.159645293	897
CchWRKY15	CchWRKY47	0.317022261	1.108089279	0.286098121	1521
CchWRKY16	CchWRKY49	0.302331684	1.165719199	0.259352067	516
CchWRKY24	CchWRKY41	0.216639899	0.519569406	0.416960461	903
CchWRKY26	CchWRKY30	0.371553064	1.3148497	0.282582157	957
CchWRKY27	CchWRKY59	0.118478255	0.510747272	0.231970411	882
CchWRKY28	CchWRKY34	0.183099966	0.320543669	0.571216914	1257
CchWRKY28	CchWRKY36	0.457413351	1.409208513	0.324588836	1140
CchWRKY29	CchWRKY38	0.115677004	0.545588838	0.212022306	2127
CchWRKY33	CchWRKY87	0.127281953	0.740333079	0.171925255	1650
CchWRKY34	CchWRKY36	0.639230897	1.494018805	0.427860008	1305
CchWRKY35	CchWRKY57	0.307717171	2.447212354	0.125741916	1317
CchWRKY51	CchWRKY54	0.138266263	0.596733594	0.231705177	933
CchWRKY52	CchWRKY55	0.131771752	0.657159381	0.200517189	771
CchWRKY53	CchWRKY57	0.144684207	0.786902711	0.183865432	1614
CchWRKY53	CchWRKY87	0.267685293	1.994005673	0.134245001	1539
CchWRKY57	CchWRKY87	0.267741492	2.200635732	0.12166552	1617
CchWRKY60	CchWRKY16	0.280446314	1.168937731	0.239915529	510
CchWRKY60	CchWRKY49	0.086801964	0.546178875	0.15892589	543
CchWRKY64	CchWRKY20	0.110126616	0.467065643	0.235784023	1422
CchWRKY66	CchWRKY83	0.2479682	1.486048308	0.166864158	1590
CchWRKY66	CchWRKY19	0.268178552	1.515606077	0.176944759	1608
CchWRKY69	CchWRKY76	0.17925063	0.634954364	0.282304745	975
CchWRKY69	CchWRKY26	0.370683982	1.732256277	0.213989112	975
CchWRKY69	CchWRKY42	0.412123887	2.583170291	0.159541896	1011
CchWRKY70	CchWRKY25	0.28522531	1.816998256	0.156976106	549
CchWRKY71	CchWRKY77	0.238302159	0.641160626	0.371673102	888
CchWRKY71	CchWRKY24	0.48900652	2.197714905	0.222506804	870

CchWRKY72	CchWRKY48	0.161111722	0.452889503	0.35574179	831
CchWRKY73	CchWRKY27	0.344432157	2.560052466	0.134541054	864
CchWRKY74	CchWRKY85	0.160330765	0.338055248	0.474273854	747
CchWRKY75	CchWRKY84	0.080421636	0.422665764	0.190272416	966
CchWRKY76	CchWRKY26	0.407183774	1.581441378	0.257476363	924
CchWRKY76	CchWRKY30	0.365536147	1.820778689	0.200758142	939
CchWRKY81	CchWRKY13	0.19103404	0.652250562	0.292884439	1560
CchWRKY82	CchWRKY14	0.175187954	0.720209179	0.243245934	915
CchWRKY83	CchWRKY19	0.106085293	0.552495128	0.192011272	1608

Table S4. Expression data of key biosynthetic genes in the pigment synthesis pathway of *C. chekiangoleosa* at different developmental stages. S1/2/3/4/5/6 refer to the six stages, P1/2/3 indicate three replicates per stage.

Gene Name	Gene ID	S1-P1	S1-P2	S1-P3	S2-P1	S2-P2	S2-P3	S3-P1	S3-P2	S3-P3	S4-P1	S4-P2	S4-P3	S5-P1	S5-P2	S5-P3	S6-P1	S6-P2	S6-P3
PSY1	Cole01G003601	18.165737	5.982007	34.797638	21.463821	14.639233	14.260851	12.666024	23.851124	13.196104	14.965017	13.205213	19.998667	12.654762	17.616936	15.248771	15.27451	17.085838	13.676265
PSY2	Cole02G001924	0.448623	0.054589	1.105091	0.314825	0.097855	0.438453	0.268487	0.756314	0.273748	0.400413	0.362431	1.822278	0.228445	0.64927	0.133729	0.30155	0.401137	0.585161
PSY3	Cole15G000116	0	0	0	0	0	0	0	0	0	0	0	0	0	0	0	0	0	0
4CL1	Cole05G000057	0	0.231362	0.216693	0.857071	0.625595	0.202493	0.813154	0.087214	0.319081	0.042435	0.22618	0.47908	0.610957	0.292896	0.313997	0.866616	1.047631	0.301049
4CL2	Cole07G004728	0	0	0	0	0	0	0	0	0	0	0.016687	0	0	0	0	0	0	0
4CL3	Cole12G002254	22.420589	17.724251	23.109499	29.234064	18.762777	25.467754	24.728691	25.026838	30.675016	27.652998	27.806618	23.546064	28.584444	31.805647	28.280779	27.65682	20.143538	24.636086
4CL4	Cole13G000656	0	0	0	0	0	0	0	0	0	0	0	0	0	0	0.05481	0	0	0
4CL5	Cole14G001409	36.927284	33.816547	37.792549	27.329422	13.391317	27.610268	20.547298	33.260262	39.745499	24.092846	45.578136	30.079517	25.020016	11.592464	20.397001	13.767173	11.757507	8.929539
4CL6	Cole14G001765	11.745212	77.256683	11.94794	8.639365	8.375998	9.664479	4.848706	11.880867	7.536934	3.194658	1.995918	6.856002	5.310436	14.910715	20.774677	22.265808	28.714111	11.507222
PAL1	Cole01G000697	8.369081	214.950027	9.937588	11.83934	6.397418	15.868849	8.240653	22.265244	15.311366	2.266757	1.322805	2.143922	5.10573	6.930376	23.332266	2.460698	1.991544	3.06315
PAL2	Cole10G002179	8.566839	162.25032	6.177239	1.747768	3.004211	4.655662	0.209453	3.557886	1.206927	0.28408	0.261566	0.411008	0.623528	0.416413	4.504637	0.360948	0.537499	1.068208
PAL3	Cole12G002301	47.205845	492.863525	87.220314	144.275024	23.902889	118.997955	226.357574	303.396942	299.749023	5.616161	12.357285	25.577915	103.672974	193.345764	306.6073	93.093353	40.13464	43.259548
C4H1	Cole03G003261	3.166244	18.291553	3.693601	5.610498	2.521039	7.739855	5.766907	10.69038	9.581511	1.350003	1.692424	2.786774	3.808578	5.049178	8.269145	3.139418	2.132745	1.968958
C4H2	Cole03G003263	0.070795	0.107261	0	0.028758	0.013423	0.09082	0.09094	0.03548	0.101242	0.040281	0	0.03044	0.028138	0.007624	0.076897	0.043025	0	0
C4H3	Cole15G002119	98.443275	813.790894	91.29142	182.70549	125.465088	185.817444	238.103439	254.402527	258.431488	82.077255	75.440338	66.992195	178.847641	240.403778	319.243713	216.859161	135.796219	134.224426
PDS1	Cole06G000881	15.635524	9.611059	15.703397	23.385706	17.099455	21.337767	16.736933	23.868458	21.416452	13.332371	13.904823	16.865763	20.295517	21.785051	19.88884	29.23176	31.456223	21.280457

Table S5. The primers used for construction of the pGADT7-CchWRKY vector.

Gene Name	Gene ID	Primers (5′ - 3′)	Enzyme Sites
CchWRKY15-F	Cole02G004361	ATGGCTAAGACCGACAAGGTCTCA	EcoR I
CchWRKY15-R		TTATGCTATGATTTGCTCTTCTTT	BamH I
CchWRKY19-F	Cole03G003319	ATGGCTTCTTCTTCAGAAATCACA	EcoR I
CchWRKY19-R		TCATCGTAGGTACGAGTCGATGAA	BamH I
CchWRKY24-F	Cole04G004808	ATGGCGGATGATAACTGGGATCTA	EcoR I
CchWRKY24-R		TCAGCCATCGCCGCCGGCAGTTGT	BamH I
CchWRKY33-F	Cole06G003067	ATGGCCCAAGGAAGAAGTGAACTC	EcoR I
CchWRKY33-R		TTAATTCCCCATAAAACTTGAATT	BamH I
CchWRKY41-F	Cole07G002227	ATGGAAGACGATTGGGATCTCCAC	EcoR I
CchWRKY41-R		TCAGCCACCGCCGGCGGCGGTGGT	BamH I
CchWRKY47-F	Cole07G004029	ATGGCTGAAAACGAAGGATCTTTG	EcoR I
CchWRKY47-R		CTATGTCAATTCTTCTTTAAACCG	BamH I
CchWRKY53-F	Cole08G001659	ATGGACAAAGGATGGGGATTAACT	EcoR I
CchWRKY53-R		TCAATTCCCTGGAAAACTACCAAT	BamH I
CchWRKY57-F	Cole08G003715	ATGGACAAAGGGTGGGGGCTCACC	EcoR I
CchWRKY57-R		TCAGTTCCCTGGAAAGCTGCCAAT	BamH I
CchWRKY66-F	Cole10G002983	ATGGCCTCCTCAGGTGGGAGTTTG	EcoR I
CchWRKY66-R		TCAGTATAGGAATGAATCGCTGAA	BamH I
CchWRKY69-F	Cole11G000153	ATGGAGAATGGTTTGAATTGGGAA	EcoR I
CchWRKY69-R		CTAATTGAAAAATCCAGGATTGTC	BamH I
CchWRKY76-F	Cole13G001467	ATGTGTACAATGGACAGTGGTTTG	EcoR I
CchWRKY76-R		TTAATAGAAATATCCAGGGGTATC	BamH I
CchWRKY83-F	Cole15G002036	ATGGCATCTTCTTCTGGGAGTTTA	EcoR I
CchWRKY83-R		TTAGTATTGCAATGACTCAAAGAA	BamH I
CchWRKY87-F	Cole00G064114	ATGGCCAAAGGAAGTGGACTCTCC	EcoR I
CchWRKY87-R		TTACTTCTCCGGAAAACTTGAATT	BamH I

Table S6. The primers used for construction of the pAbAi-*PSY*/*4CL*/*PAL*/*C4H*/*PDS* vector.

Gene Name	Gene ID	Primers (5′ - 3′)	Enzyme Sites
PSY1-F	Cole01G003601	AGTTCCGACCAAATCAGTAAAACA	Sac I
PSY1-R		TTTCTGCAAATTGGGAGTGACTAA	BamH I
PSY2-F	Cole02G001924	ATGTAATTTAGGCCTTGTTTGGGT	Sac I
PSY2-R		CTGGGATTTCTGTACAATGAACCA	BamH I
PSY3-F	Cole15G000116	GTTCTTATGCAACAGACAAAACCC	Sac I
PSY3-R		AAATAGTGTACACCCTCCTCCTAC	BamH I
4CL1-F	Cole05G000057	GCATCACTCTGGTAAAGGTAAACA	Sac I
4CL1-R		GACTCCAACTTTTCTTCTCTAGGC	BamH I
4CL2-F	Cole07G004728	AGTGATTAATGATGGTGTTGTGCT	Sac I
4CL2-R		TAATACGCCTAAGACCATCTCCAA	BamH I
4CL3-F	Cole12G002254	TTCCACGAATTTGTCTAGCGATTT	Sac I
4CL3-R		ATTTTGCAGAGGGTTTGATTTTGG	BamH I
4CL4-F	Cole13G000656	ATATCAATTAGTGCCCAAATCCCC	Sac I
4CL4-R		GTGAGAAAGACCAGAAAACTAGGG	BamH I
4CL5-F	Cole14G001409	CATCTCCAACGGTTACTCAGATTT	Sac I
4CL5-R		TAGAGACAGAGAGACAGAGAGAGT	BamH I
4CL6-F	Cole14G001765	CCATTGCAATCGTGATCTAAAACC	Sac I
4CL6-R		TTGTTAGGGGATTGAATTGGTGAG	BamH I
PAL1-F	Cole01G000697	AGTGATGCTCTTCTTGTTGCTAAA	Sac I
PAL1-R		CCGAGGTGTTAGTTGAGAAATTCA	BamH I
PAL2-F	Cole10G002179	TCACCTTGGACACAGCTAACTATA	Sac I
PAL2-R		AGTAGGTAAGCATAAAGGGGAGTT	BamH I
PAL3-F	Cole12G002301	TTTATGCAAGGGATAGTCTTAGGC	Sac I
PAL3-R		CATGACTCTGTCCCATTTCCAAAT	BamH I
C4H1-F	Cole03G003261	CTTTTGTTGTCTTGAGGTGAGAGT	Sac I
C4H1-R		AAACCTAAACTCAAGCCTAAACCC	BamH I
C4H2-F	Cole03G003263	CAACAAATTTAGGAAACAGCCGTG	Sac I
C4H2-R		GTGTTTGATTGGGGAGATAAGAGG	BamH I
C4H3-F	Cole15G002119	AAGTTTGGCAATCTTCTATGACGT	Sac I
C4H3-R		GAATTTGTGTATGCCAATCGTCTG	BamH I
PDS1-F	Cole06G000881	CTGCTTCTTCTTCTCTCTCTCTCT	Sac I
PDS1-R		TCACACCTGAATTTCTAAACCTGG	BamH I
